# Can metaphyseal variations in the distal femurs and proximal tibias be distinguished from classic metaphyseal lesions?

**DOI:** 10.1007/s00247-025-06398-w

**Published:** 2025-10-01

**Authors:** Boaz Karmazyn, Christopher L. Newman, Megan B. Marine, Matthew R. Wanner, Jared R. Shields, Lisa R. Delaney, Scott D. Steenburg, Alexander G. Boutselis, Jordan H. Cuskaden, Eric D. Westin, Marrisa J. Luoma, S. Gregory Jennings, George J. Eckert, Ralph A. Hicks

**Affiliations:** 1 Department of Radiology and Imaging Sciences, Riley Hospital for Children at IU Health, Indiana University School of Medicine, Indianapolis, United States; 2 Department of Radiology and Imaging Sciences, Indiana University School of Medicine, Indianapolis, United States; 3 Department of Radiology, Mayo Clinic, Phoenix, United States; 4 Department of Pediatrics, Section of Child Protection Programs, Indiana University School of Medicine, Riley Hospital for Children, Indianapolis, United States; 5 Department of Biostatistics and Health Data Science, Indiana University School of Medicine, Indianapolis, United States

**Keywords:** Classic metaphyseal lesion, Child abuse, Femur, Fracture, Infant, Metaphyseal variation, Skeletal survey radiography, Tibia

## Abstract

**Background:**

Classic metaphyseal lesions (CMLs) are considered specific for child abuse, but the reliability of radiologists in distinguishing CMLs from metaphyseal variations is unclear.

**Objective:**

To evaluate the diagnostic performance of pediatric and adult emergency radiologists in differentiating CMLs from metaphyseal variations in the knees.

**Materials and methods:**

We retrospectively reviewed distal femur and proximal tibia radiographs in children under 1 year of age who underwent skeletal surveys for suspected child abuse. A consensus diagnosis for CMLs and metaphyseal variations—serving as the ground truth—was established by two pediatric radiologists. The CML group comprised children diagnosed with abuse and confirmed CMLs. The metaphyseal variation group included children not diagnosed with abuse, who exhibited metaphyseal variations and had either no fractures or only an isolated skull fracture. Radiographs were trimmed to exclude other injuries. Four pediatric and four adult radiologists reviewed anonymized studies and categorized each case as CML, metaphyseal variation, normal, or indeterminate, with confidence levels (high, moderate, low). We analyzed diagnoses with moderate or high confidence. Interobserver agreement was assessed using kappa statistics.

**Results:**

There were 44 children with CMLs (10 initial, 7 follow-up, 27 initial and follow-up) and 22 with metaphyseal variations (10 initial, 7 follow-up, 5 initial and follow-up). Metaphyseal fragmentation was the most common variation, identified in 249 of 344 femurs (72.4%, 95% CI 67.3-77.0%) and 60 of 69 tibias (87.0%, 76.7-93.9%). Fragmentations were most frequently located in the posterior or medial metaphysis, or both, in 238 of 249 femurs (95.6%, 92.2-97.8%) and 60 of 69 tibias (87.0%, 76.7-93.9%).

In the CML group, 33 of 114 initial CML diagnoses (28.9%, 20.8-38.2%) were read on follow-up as either metaphyseal variation (*n* = 17) or normal (*n* = 16). In contrast, in the metaphyseal variation group, only one follow-up case was diagnosed as a CML; the remainder were diagnosed on follow-up as metaphyseal variation (*n* = 24).

Diagnostic performance for CML demonstrated high specificity (90.9%, 85.6-94.7%) and positive predictive value (95.6%, 93.0-97.5%), with moderate accuracy (79.3%, 75.9-82.4%), sensitivity (74.9%, 70.8-78.8%), and negative predictive value (57.6%, 51.5-63.5%). Interobserver agreement was substantial, with a mean kappa of 0.61 (range 0.45–0.84).

**Conclusion:**

Radiologists demonstrated substantial agreement and high specificity in distinguishing CMLs from metaphyseal variations. Metaphyseal fragmentation was the most common variation and was uncommonly diagnosed as CML on follow-up.

**Supplementary Information:**

The online version contains supplementary material available at 10.1007/s00247-025-06398-w.

## Introduction

One of the unique challenges in evaluating fractures in children is to differentiate between normal variation and fracture [1]. This is especially critical when evaluating radiographs for child abuse. Missing fractures can have disastrous consequences and may lead to continued trauma to the child, handicap, or even death [2]. Alternatively, overdiagnosis of injuries suggestive of child abuse can lead to unnecessary emotional distress and the removal of the child from the home. It is therefore critical for radiologists to recognize metaphyseal variations.

Classic metaphyseal lesions (CML) include corner fractures and bucket handle fractures which are distinctive radiologic findings and are highly specific for child abuse [3–7]. However, certain normal metaphyseal variations in infancy should be carefully distinguished from the CML [8–10].

Anatomically, the periphysis is the region of the growth plate that incorporates the groove zone of Ranvier and the ring of Lacroix (all of which surround the physis and adjacent primary spongiosa) [11]. The appearance of the periphysis varies with the anatomical region and radiographic projection. Two principal radiographic patterns, step-off and spur, should be recognized as normal anatomical structures [10]. Metaphyseal fragmentation, another variation, is localized to the distal femur and proximal tibia, and was found to be associated with physiological bowing in children older than 15 months of age [9]. At our institution, we have also observed this variation in younger children with a low suspicion for nonaccidental trauma.

This study aims to assess whether radiologists can accurately distinguish metaphyseal variations from CMLs.

## Materials and methods

This study was approved by the institutional review board with a waiver of consent granted. This was a retrospective review of radiographic images of distal femurs and proximal tibias in children up to 1 year of age who underwent a skeletal survey for suspected child abuse.

Using our institution’s radiology information system (RIS), a pediatric radiologist with 28 years of experience post-fellowship, who did not participate in the imaging evaluation, retrospectively retrieved all skeletal surveys performed for evaluation of child abuse from 2007 to 2023. Two groups of patients were identified. The first group included children with a diagnosis of child abuse and reports of distal femur or proximal tibia CMLs. The second group included children without a diagnosis of child abuse and a reported distal femur or proximal tibial metaphyseal variation, with either no fractures or isolated accidental skull fracture, as isolated skull fracture has been found to be associated with a low risk for child abuse [12].

For the CML group, the RIS was searched using the keywords of “CML,” “classic metaphyseal lesion,” “corner fracture,” or “bucket handle fracture.” For the metaphyseal variation group, the keywords were “irregularity,” “step off,” “beak,” “spur,” “fragmentation,” and “variation.”

### Radiograph selection and preparation

Initial and follow-up radiographs (if available) were included. From the skeletal surveys, the anteroposterior and lateral Digital Imaging and Communications in Medicine (DICOM) radiographs of the femurs and tibias were selected and anonymized. To minimize potential bias from other injuries visible on the radiographs, all images were trimmed by an experienced radiologic technologist under the supervision of a pediatric radiologist who was not involved in the image evaluation. Trimming was performed using the Santa DICOM Editor (Santsoft, Athens) to isolate the metaphysis of the distal femur or proximal tibia, or both —depending on which structures were included in the original radiograph.

### Medical record review

Two child abuse pediatricians, who were not involved in image interpretation, reviewed the electronic medical record (EMR) and documented the following: demographic information, history of prematurity, underlying medical conditions, the original radiologist’s interpretation of distal femur and proximal tibia findings, other injuries noted in the skeletal survey, additional imaging obtained to evaluate for abusive injuries, and the presence of physical injuries such as bruising, burn injuries, other skin injuries, retinal bleeding, subconjunctival hemorrhage, and torn frenula.

The reports of neuroimaging studies were reviewed for the presence of subdural hematoma, parenchymal contusion or tear, parenchymal bleeding, and hypoxic-ischemic changes. If there was no history or medical conditions to adequately explain them, the presence of at least one of these findings was considered a sign of high likelihood of abusive head trauma.

The child abuse pediatrician’s report was reviewed and categorized as positive, negative, or indeterminate for the diagnosis of physical abuse. Children with an indeterminate diagnosis of child abuse were excluded.

### Exclusion criteria

Patients with metabolic bone disease or skeletal dysplasia were excluded. Additionally, cases were excluded if the radiology report indicated a CML, but the child was not diagnosed with child abuse, or if the report described a metaphyseal variation in a child with either an intermediate or positive diagnosis of child abuse. Radiographs were also excluded if imaging of the distal femurs and proximal tibias was limited to a single view, obscured by casting material or intraosseous lines, or demonstrated bony or soft tissue post-traumatic abnormalities that could not be adequately trimmed. Finally, patients for whom a consensus diagnosis of either CML or metaphyseal variation could not be reached were excluded from the study.

### A reference standard for diagnosis of CML and metaphyseal variation

A consensus diagnosis, serving as the ground truth, was established by two pediatric radiologists with 19 years and 28 years of post-fellowship experience respectively, who were unblinded to clinical and imaging follow-up. Based on this consensus, cases were categorized into two groups: those with CML and those with metaphyseal variations.

### Radiograph review

DICOM images were reviewed independently by eight radiologists using a diagnostic high-resolution monitor on the Synapse PACS system (FUJIFILM Medical Systems, Lexington, MA). All evaluations were performed in a randomized order and blinded to clinical information. To prevent paired comparison, initial and follow-up radiographs were separated and randomized, ensuring that radiologists could not view corresponding studies concurrently. Four reviewers were pediatric radiologists with 2 years, 5 years, 13 years, and 19 years of experience. Four were adult emergency radiologists with 5 years, 16 years, 16 years, and 23 years of experience. Data collection and management were conducted using a secure, web-based application (REDCap; Vanderbilt University, Nashville, TN).

For each metaphysis, the radiologist selected one of the following options: normal, variation, CML, or indeterminate. If normal, variation, or CML was selected, the radiologist assigned a level of low, moderate, or high confidence.

**Fig. 1:**
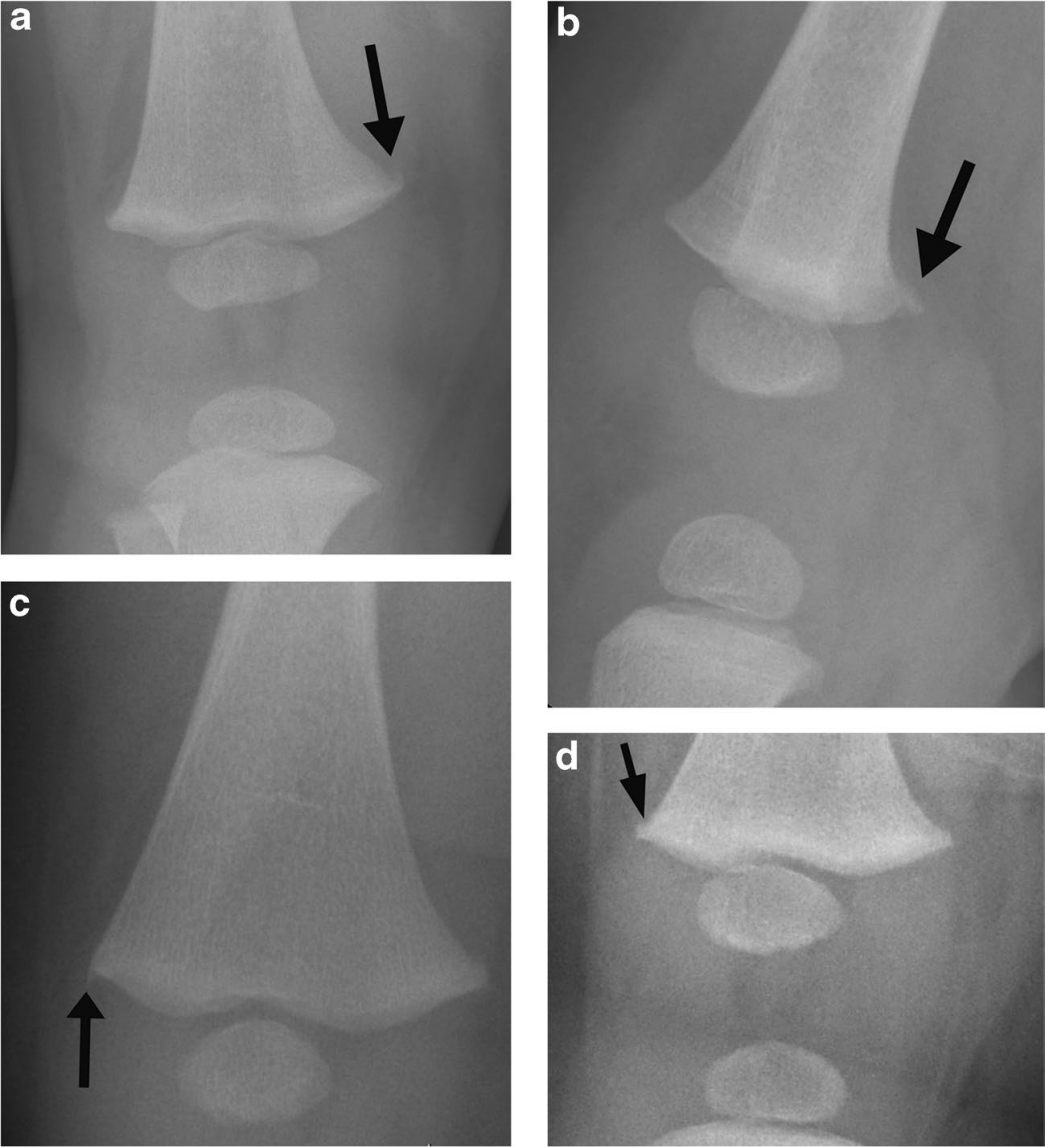
Metaphyseal variations a. Metaphyseal fragmentation variation in a 3 month-old girl. Anteroposterior (AP) radiograph of the right knee demonstrates bony fragmentation extending proximally from the distal medial femoral metaphyseal corner (arrow). b. Lateral radiograph of the right knee in the same patient shows corresponding posterior metaphyseal fragmentation (arrow). c. Metaphyseal spur variation in a 4 month-old boy. AP radiograph of the right knee demonstrates a discrete longitudinal projection of bone (arrow) that is continuous with the cortex and extends beyond the metaphyseal margin. d. Metaphyseal step off variation in a 7 month-old boy. AP radiograph of the right knee demonstrates an acute cortical angulation at the junction of the distal lateral metaphysis and the physis (arrow)

For diagnosing CML, radiologists identified corner or bucket-handle fractures (any healing stage), corner deformity, subphyseal lucency, or metaphyseal irregularity with a rough metaphyseal–physeal margin. Metaphyseal variation (Fig. 1) is diagnosed by the presence of step-off (acute discrete distal metaphyseal angulation), spur (longitudinal thin cortical projection beyond the metaphyseal edge), or fragmentation (metaphyseal corner fragmentation oriented along the shaft), with the specific location (medial, lateral, anterior, or posterior) noted. The location is described as affecting either the entire metaphysis or at least one of the following locations: medial, lateral, anterior, or posterior metaphysis.

In cases where both a metaphyseal variant and a CML were present, only the CML is included in the analysis.

Before formally reviewing images, a pilot training session was conducted using 11 cases not meeting the study’s inclusion criteria and excluded from the final cohort. These cases included six metaphyseal variations, four CMLs, and one indeterminate metaphyseal irregularity. The annotated cases were presented to the readers to familiarize them with the spectrum of appearances and reinforce the classification criteria.

#### Statistical analysis

Fisher’s exact tests and two-sample *t*-tests were used to compare the CML and metaphyseal variation groups for differences in patient characteristics. Agreement between radiologists in diagnoses with moderate and high confidence was evaluated using crosstabs, percentage agreement, and kappa statistics. Kappa values of 0.10–0.20 were considered as slight, 0.21–0.40 as fair, 0.41–0.60 as moderate, 0.61–0.80 as substantial, and 0.81–0.99 as near perfect agreement [13]. A bootstrap analysis with 10,000 iterations was used to test the difference in kappa statistics between pediatric and adult radiologists.

For sensitivity and specificity calculations, we evaluated diagnoses with moderate and high confidence. A true positive diagnosis was defined as follows: a diagnosis of CML was considered correct for children in the CML group, and a diagnosis of metaphyseal variation was considered correct for children in the metaphyseal variation group. Generalized estimating equation models (GEEs) for binary outcomes were used to obtain combined estimates across all radiologists for accuracy, sensitivity, specificity, positive predictive value (PPV), and negative predictive value (NPV). The GEEs accounted for correlations among readings from multiple radiologists for each patient. GEEs were then used to compare pediatric and adult emergency radiologists for differences in the percentages of correct moderate or high confidence interpretations.

Using all confidence diagnoses levels, GEEs were also used to compare patients with metaphyseal variation and CML for differences in the frequencies of radiographic signs typically associated with metaphyseal variation (metaphyseal step off, beak, spur, fragmentation) and radiographic signs typically associated with CML (corner fracture, bucket-handle fracture, subphyseal lucency, metaphyseal irregularity).

GEEs were used to compare confidence levels (low confidence and indeterminate diagnoses versus moderate and high confidence diagnoses) between pediatric and adult emergency radiologists.

## Results

### Patient population

**Fig. 2:**
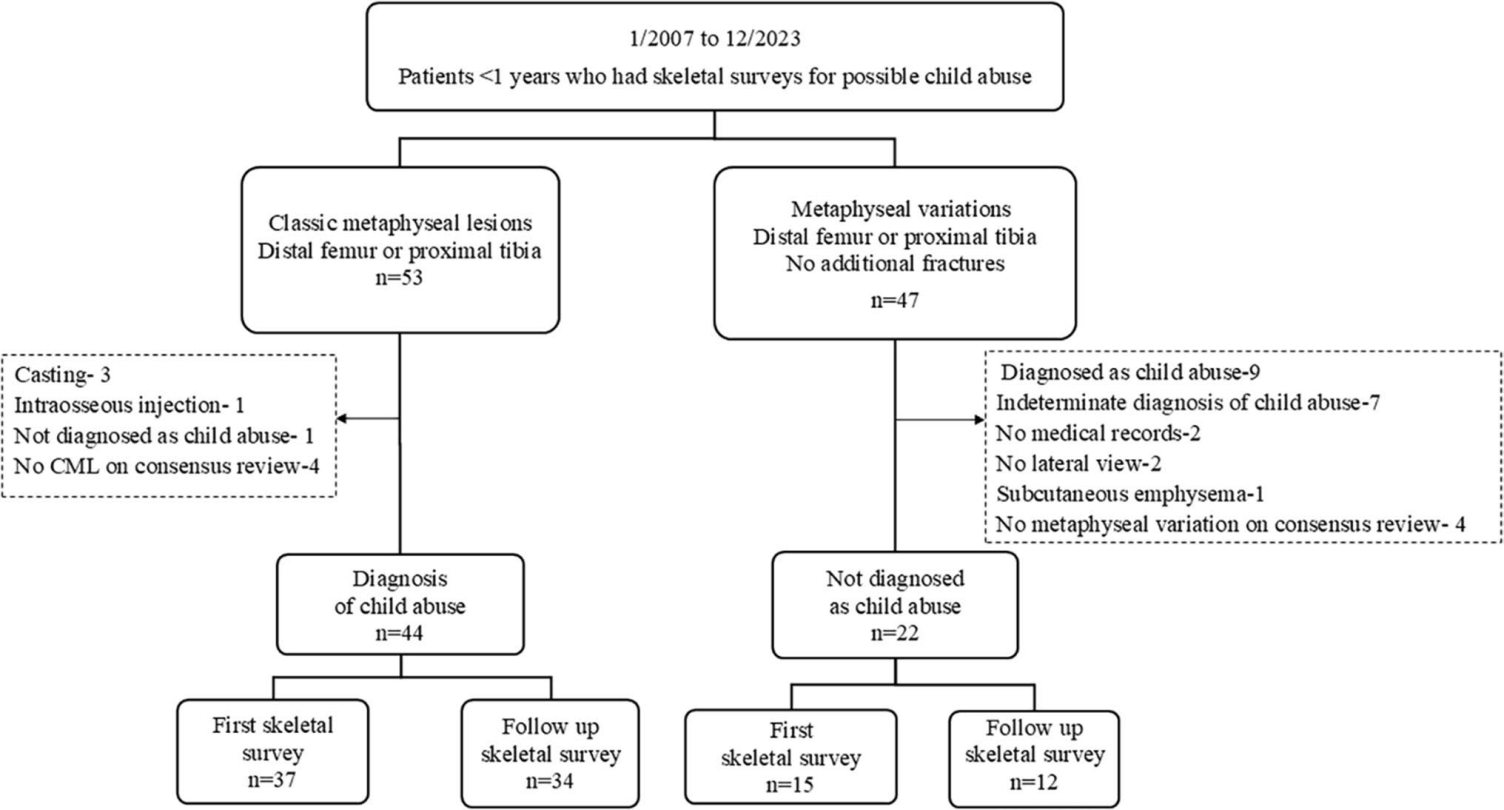
Flow chart of patients with classic metaphyseal lesions and metaphyseal variations

Figure 2 illustrates patient selection. A total of 53 children were initially reported to have CMLs involving the distal femur or proximal tibia. Nine children were excluded for the following reasons: presence of casting material on radiographs (*n* = 3), intraosseous needle evident on imaging (*n* = 1), lack of a diagnosis of child abuse (*n* = 1), and no consensus diagnosis of CML (*n* = 4). The final cohort included 44 children with radiology reports of CML, who underwent 71 skeletal surveys (37 initial and 34 follow-up studies).

Similarly, 47 children had reports indicating metaphyseal variation in the distal femur or proximal tibia. Of these, 25 were excluded due to a definite (*n* = 9) or indeterminate (*n* = 7) diagnosis of child abuse, no medical records (*n* = 2), absence of a lateral view (*n* = 2), presence of subcutaneous emphysema (*n* = 1), and no consensus diagnosis of metaphyseal variation (*n* = 4). The final metaphyseal variation group included 22 children who underwent a total of 27 skeletal surveys (15 initial and 12 follow-ups).

It was possible to trim all radiographs, such that each imaging set (initial or follow-up) included eight radiographs: trimmed AP and lateral views of the bilateral femurs and tibias.

Children in the CML group (mean age 3 months, range 1–12 months) were significantly younger than those in the metaphyseal variation group (mean age 6 months, range 1–12 months; *P* < 0.001). The CML group comprised 29 males (65.9%, 95% CI 50.1-79.5%) and 15 females (34.1%, 20.5-49.9%), while the metaphyseal variation group included 17 males (77.3%, 54.6-92.2%) and five females (22.7%, 7.8-45.4%), with no statistically significant sex distribution difference between groups (*P* = 0.405). Supplementary Table S1 summarizes the injury characteristics of children with CMLs and those with metaphyseal variations.

### Diagnostic accuracy of radiologists (moderate to high confidence)

Table 1 summarizes diagnostic performance. Overall, radiologists demonstrated moderate diagnostic accuracy (79.3%, 75.9-82.4%) and sensitivity (74.9%, 70.8-78.8%), with high specificity (90.9%, 85.6-94.7%) and PPV (95.6%, 93.0-97.5%) in diagnosis of CMLs and distinguishing them from metaphyseal variations. The NPV was moderate at 57.6% (51.5-63.5%).

**Table 1:** Accuracy of radiologists to diagnose classic metaphyseal lesions, % (95% CI)

Radiologist	Accuracy	Sensitivity	Specificity	PPV	NPV
All	79.3% (75.9-82.4%)	74.9% (70.8-78.8%)	90.9% (85.6-94.7%)	95.6% (93.0-97.5%)	57.6% (51.5-63.5%)
Ped-All	*83.6% (79.1-87.4%)	*79.3% (73.%−84.3%)	94.6% (87.8-98.2%)	97.4% (94.1-99.2%)	*64.0% (55.3-72.0%)
Adult-All	74.8% (69.6-79.5%)	70.4% (64.1-76.3%)	86.7% (77.5-93.2%)	93.6% (88.9-96.8%)	51.4% (42.8-60.0%)
Ped-1	94.3% (86.0-98.4%)	92.2% (81.1-97.8%)	100% (82.4-100%)	100% (92.5-100%)	82.6% (61.2-95.0%)
Ped-2	89.4% (80.8-95.0%)	85.2% (73.8-93.0%)	100% (85.8-100%)	100% (93.2-100%)	72.7% (54.5-86.7%)
Ped-3	75.3% (64.7-84.0%)	66.7% (53.3-78.3%)	96.0% (79.6-99.9%)	97.6% (87.1-99.9%)	54.5% (38.8-69.6%)
Ped-4	77.5% (67.4-85.7%)	75.4% (63.1-85.2%)	83.3% (62.6-95.3%)	92.5% (81.8-97.9%)	55.6% (38.1-72.1%)
Adult-1	65.2% (54.3-75.0%)	59.4% (46.4-71.5%)	80.0% (59.3-93.2%)	88.4% (74.9-96.1%)	43.5% (28.9-58.9%)
Adult-2	72.7% (61.4-82.3%)	63.6% (49.6-76.2%)	95.5% (77.2-99.9%)	97.2% (85.5-99.9%)	51.2% (35.1-67.1%)
Adult-3	86.8% (76.4-93.8%)	90.4% (79.0-96.8%)	75.0% (47.6-92.7%)	92.2% (81.1-97.8%)	70.6% (44.0-89.7%)
Adult-4	77.2% (66.4-85.9%)	71.2% (57.9-82.2%)	95.0% (75.1-99.9%)	97.7% (87.7-99.9%)	52.8% (35.5-69.6%)

PPV,  positive predictive value; NPV, negative predictive value; Ped-, pediatric radiologists; Adult-, adult emergency radiologists*Significant difference between pediatric and adult radiologists (all P < 0.005).

Pediatric radiologists outperformed their adult radiology counterparts, with significantly higher accuracy (83.6% [79.1-87.4%] vs. 74.8% [69.6-79.5%], *P* < 0.001), sensitivity (79.3% [73.6-84.3%] vs. 70.4% [64.1-76.3%], *P* = 0.002), and NPV (64.0% [55.3-72.0%] vs. 51.4% [42.8-60.0%], *P* < 0.001). No significant differences were found in specificity (94.6% [87.8-98.2%] vs. 86.7% [77.5-93.2%], *P* = 0.104) or PPV (97.4% [94.1-99.2%] vs. 93.6% [88.9-96.8%], *P* = 0.106).

Diagnostic performance was higher on initial radiographs compared to follow-up radiographs, with significantly higher accuracy (*P* = 0.003), sensitivity (*P* < 0.001), and NPV (*P* = 0.030). However, specificity (*P* = 0.147) and PPV (*P* = 0.488) did not differ significantly between visits.

### Change between initial and follow-up radiographs (moderate to high confidence)

In the CML group, 27 of the 44 children had both initial and follow-up radiographs (Table 2). In total, 216 initial and follow-up studies were interpreted by the eight radiologists. Of these, 143 studies were read with moderate or high confidence. Overall, radiologists identified CML with moderate or high confidence in 114 out of 143 studies (79.7%, 72.2-86.0%). An additional nine children were diagnosed as having CMLs on follow-up radiographs: four originally diagnosed as metaphyseal variation and five as normal.

**Table 2:** Changes in moderate and high confidence diagnoses of classic metaphyseal lesions (*CMLs*) and metaphyseal variation groups from initial to follow-up reads by all eight radiologists, *N* (%, 95% CI)

		**Follow-up**			
Radiologist		CML	Metaphyseal variation	Normal	Total
	**Initial**				
**CML patients (*n* = 27 children, 143 moderate/high confidence reads)**					
All	CML	81 (71.1%, 61.8-79.2%)	17 (14.9%, 8.9-22.8%)	16 (14.0%, 8.2-21.8%)	114 (79.7%, 72.2-86.0%)
	Metaphyseal variation	4 (21.1%, 6.1-45.6%)	8 (42.1%, 20.3-66.5%)	7 (36.8%, 16.3-61.6%)	19 (13.3%, 8.2-20.0%)
	Normal	5 (50.0%, 18.7-81.3%)	1 (10.0%, 0.3-44.5%)	4 (40.0%, 12.2-73.8%)	10 (7.0%, 3.4-12.5%)
	Total	90 (62.9%, 54.5-70.9%)	26 (18.2%, 12.2-25.5%)	27 (18.9%, 12.8-26.3%)	143
Pediatric radiologists	CML	47 (73.4%, 60.9-83.7%)	10 (15.6%, 7.8-26.9%)	7 (10.9%, 4.5-21.2%)	64 (85.3%, 75.3-92.4%)
	Metaphyseal variation	1 (12.5%, 0.3-52.7%)	5 (62.5%, 24.5-91.5%)	2 (25.0%, 3.2-65.1%)	8 (10.7%, 4.7-19.9%)
	Normal	2 (66.7%, 9.4-99.2%)	0 (0%, 0-70.8%)	1 (33.3%, 0.8-90.6%)	3 (4.0%, 0.8-11.2%)
	Total	50 (66.7%, 54.8-77.1%)	15 (20.0%, 11.6-30.8%)	10 (13.3%, 6.6-23.2%)	75
Adult radiologists	CML	34 (68.0%, 53.3-80.5%)	7 (14.0%, 5.8-26.7%)	9 (18.0%, 8.6-31.4%)	50 (73.5%, 61.4-83.5%)
	Metaphyseal variation	3 (27.3%, 6.0-61.0%)	3 (27.3%, 6.0-61.0%)	5 (45.5%, 16.7-76.6%)	11 (16.2%, 8.4-27.1%)
	Normal	3 (42.9%, 9.9-81.6%)	1 (14.3%, 0.4-57.9%)	3 (42.9%, 9.9-81.6%)	7 (10.3%, 4.2-20.1%)
	Total	40 (58.8%, 46.2-70.6%)	11 (16.2%, 8.4-27.1%)	17 (25.0%, 15.3-37.0%)	68
**Metaphyseal variation patients (*n* = 5 children, 25 moderate/high confidence reads)**					
All	CML	0	0	0	0 (0%, 0-13.7%)
	Metaphyseal variation	1 (4.3%, 0.1-21.9%)	22 (95.7%, 78.1-99.9%)	0	23 (92.0%, 74.0-99.0%)
	Normal	0 (0%, 0-84.2%)	2 (100%, 15.8-100%)	0	2 (8.0%, 1.0-26.0%)
	Total	1 (4.0%, 0.1-20.4%)	24 (96.0%, 79.6-99.9%)	0 (0%, 0-13.7%)	25
Pediatric radiologists	CML	0	0	0	0 (0%, 0-23.2%)
	Metaphyseal variation	0	14 (100%, 76.8-100%)	0	14 (100%, 76.8-100%)
	Normal	0	0	0	0 (0%, 0-23.2%)
	Total	0 (0%, 0-23.2%)	14 (100%, 76.8-100%)	0 (0%, 0-23.2%)	14
Adult radiologists	CML	0	0	0	0 (0%, 0-28.5%)
	Metaphyseal variation	1 (11.1%, 0.3-48.2%)	8 (88.9%, 51.8-99.7%)	0	9 (81.8%, 48.2-97.7%)
	Normal	0 (0%, 0-84.2%)	2 (100%, 15.8-100%)	0	2 (18.2%, 2.3-51.8%)
	Total	1 (9.1%, 0.2-41.3%)	10 (90.9%, 58.7-99.8%)	0 (0%, 0-28.5%)	11

**Fig. 3:**
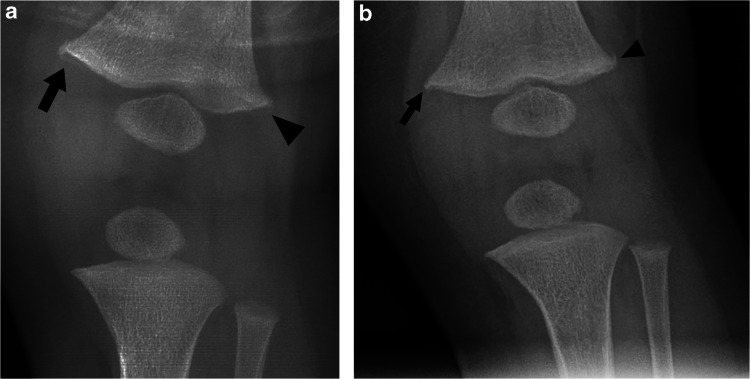
A subtle classic metaphyseal lesion (CML) in a 7 month-old boy with a challenging follow-up radiograph mimicking a metaphyseal variation. a. Anteroposterior (AP) radiograph shows a distal left femoral subtle bucket handle fracture in the medial (arrow) and lateral metaphysis (arrowhead) suggestive of CML. b. Fifteen-day follow up AP radiograph shows distal femur medial corner irregularity (arrow) and lateral metaphysis spur (arrowhead). Six radiologists independently reviewed the initial and follow-up radiographs with moderate to high confidence, blinded to clinical history. The three pediatric radiologists diagnosed a CML initially and a metaphyseal variation on follow-up, while the three adult emergency radiologists diagnosed a metaphyseal variation initially; on follow-up, two diagnosed again metaphyseal variation and one read the study as normal

Notably, 33 of 114 studies (28.9%, 20.8-38.2%) initially diagnosed with CML were reclassified on follow-up as having metaphyseal variation (*n* = 17) or a normal metaphysis (*n* = 16) (Fig. 3).

**Fig. 4:**
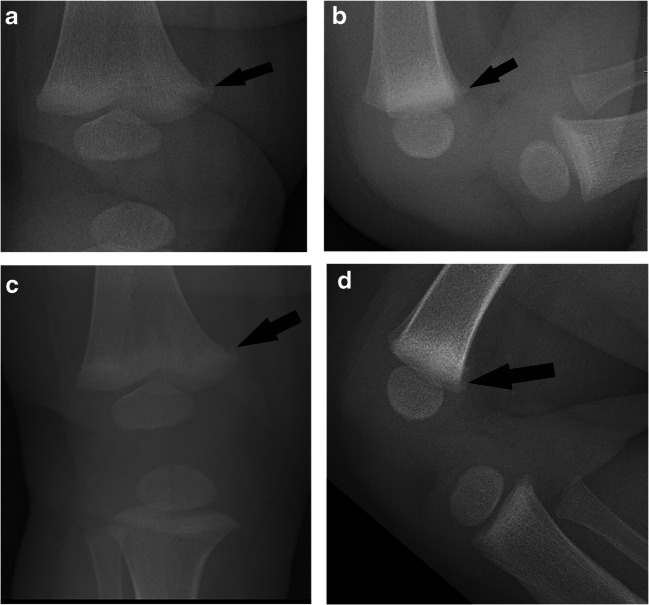
Distal right femur metaphyseal fragmentation in a 5-month-old boy. a Anteroposterior (AP) radiograph shows medial metaphyseal fragmentation (arrow). b Lateral radiograph shows posterior metaphyseal fragmentation (arrow). c Fourteen-day follow-up AP radiograph shows no interval change in the medial metaphyseal fragmentation (arrow). d Fourteen-day follow-up lateral radiograph shows no interval change in the posterior metaphyseal fragmentation (arrow). All radiologists reviewing separately the initial and follow-up radiographs blinded to clinical history diagnosed metaphyseal variation with moderate to high confidence on both the initial and follow-up radiographs. Seven radiologists diagnosed metaphyseal fragmentation both in the initial and follow-up radiographs, and one radiologist as metaphyseal step off

In the metaphyseal variation group, five of the 22 children had both initial and follow-up radiographs. Only one follow-up study was interpreted as CML, by an adult radiologist. All others were interpreted as metaphyseal variation on follow-up (*n* = 24, Fig. 4).

### Location-based analysis of CMLs and metaphyseal variations (all confidence levels)

Tables S2 and S3 summarize the locations of metaphyseal variants and CMLs in the femurs and tibias. In the CML group, abnormalities were 1.4 times more common in the femur than in the tibia. CMLs were identified in 392 of 568 (69.0%, 65.0-72.8%) femurs and 275 of 568 (48.4%, 44.2-52.6%) tibias. In the metaphyseal variation group, abnormalities were 5.2 times more common in the femurs as compared with the tibias. Variants were identified in 162 of 216 (75.0%, 68.7-80.6%) femurs and 31 of 216 (14.4%, 10.0-19.8%) tibias.

Among metaphyseal variants, fragmentation was most common: 249 of 344 femoral lesions (72.4%, 67.3-77.0%) and 60 of 69 tibial lesions (87.0%, 76.7-93.9%). This was followed by metaphyseal spurs, with 60 of 344 femurs (17.4%, 13.6-21.9%) and three of 69 tibias (4.4%, 0.9-12.2%), and metaphyseal step-off in 35 femurs (10.2%, 7.2-13.9%) and six tibias (8.7%, 3.3-18.0%).

Metaphyseal fragmentation demonstrated a highly consistent distribution, occurring in the posterior and/or medial metaphyses in 238 of 249 femurs (95.6%, 92.2-97.8%) and all 60 tibias (100%, 94.0-100%).

Metaphyseal spurs were mostly identified in the anterior and/or lateral metaphyses in 49 of 60 femurs (81.7%, 69.6-90.5%). In contrast, the three spurs observed in the tibias were distributed across variable locations.

Metaphyseal step-offs, when confined to a single location, were nearly equally distributed between the medial (11/35, 31.4%, 16.9-49.3%) and lateral metaphyses of the femur (12/35, 34.3%, 19.1-52.2%). In seven of the 35 cases (20%, 8.4-36.9%), the step-off involved only the posterior metaphysis, whereas in four of 35 cases (11.4%, 3.2-26.7%), the step-off involved the posterior metaphysis in combination with either the medial or lateral side. One case demonstrated the involvement of both medial and lateral metaphyses. In the tibia, all six step-off cases were located in the medial metaphysis.

CMLs signs involved the entire metaphysis or at least three metaphyseal locations in 394 of 639 femurs (61.7%, 57.8-65.5%) and 346 of 489 tibias (70.8%, 66.5-74.8%). In contrast, metaphyseal variations involved the entire metaphysis or ≥ 3 locations in only one of 344 femurs (0.3%, < 0.1-1.6%) and one of 69 tibias (1.4%, < 0.1-7.8%).

### Confidence comparison

There was no difference (*P* = 0.088) in the proportion of low-confidence or indeterminate diagnoses versus moderate or high-confidence between pediatric and adult radiologists, occurring in 63 of 392 (16.1%, 12.6-20.1%) and 79 of 392 (20.2%, 16.3-24.5%) of the cases, respectively.

### Agreement between radiologists (moderate and high confidence diagnoses)

The average agreement between radiologists was substantial (kappa = 0.61), ranging from moderate to substantial (kappa of 0.45 to 0.84) between radiologist pairs (Table 3). The most experienced pediatric radiologists (pair 1 and 2) demonstrated the highest agreement (kappa = 0.84, 0.72–0.96). There was a statistically significant difference in the kappa statistics between pediatric and adult radiologists (*P = *0.040), with a mean difference of 0.10 (95% CI 0.01 to 0.20).

**Table 3:** Agreement between radiologists on diagnosis of classic metaphyseal lesions and metaphyseal variations

			Kappa (95% CI)		
Radiologist	Pair	All	Initial		Follow-up
Pediatric	1 and 2	0.84 (0.72 to 0.96)	0.87 (0.72 to 1.00)		0.80 (0.61 to 0.99)
	1 and 3	0.59 (0.40 to 0.77)	0.57 (0.31 to 0.83)		0.58 (0.30 to 0.85)
	1 and 4	0.63 (0.45 to 0.81)	0.56 (0.26 to 0.85)		0.66 (0.41 to 0.90)
	2 and 3	0.68 (0.53 to 0.83)	0.76 (0.56 to 0.96)		0.58 (0.35 to 0.81)
	2 and 4	0.61 (0.46 to 0.76)	0.64 (0.43 to 0.86)		0.55 (0.33 to 0.77)
	3 and 4	0.56 (0.41 to 0.72)	0.46 (0.23 to 0.69)		0.62 (0.40 to 0.83)
	Average	0.65	0.64		0.63
Adult	1 and 2	0.53 (0.38 to 0.69)	0.52 (0.30 to 0.74)		0.52 (0.30 to 0.75)
	1 and 3	0.50 (0.31 to 0.69)	0.42 (0.16 to 0.68)		0.56 (0.29 to 0.84)
	1 and 4	0.45 (0.29 to 0.61)	0.46 (0.22 to 0.69)		0.41 (0.18 to 0.64)
	2 and 3	0.60 (0.41 to 0.78)	0.48 (0.20 to 0.77)		0.65 (0.39 to 0.91)
	2 and 4	0.65 (0.49 to 0.81)	0.50 (0.25 to 0.75)		0.77 (0.57 to 0.97)
	3 and 4	0.58 (0.38 to 0.78)	0.58 (0.30 to 0.86)		0.56 (0.26 to 0.85)
	Average	0.55	0.49		0.58
All	Average	0.61	0.59		0.61

## Discussion

Our study demonstrates that CMLs can be distinguished from metaphyseal variations with high specificity and positive predictive values, and that metaphyseal fragmentation is the most common variation, predominantly occurring in the distal femur.

Few studies exist on metaphyseal variations that mimic CMLs. In a study by Eide et al. on 408 children under 2 years old who were admitted to the emergency department for suspected trauma, a metaphyseal collar was identified in 16.3% and irregular metaphysis variation in 8.6% of cases [5]. Kleinman et al. found step-off variation in 3% of the distal femurs in a study of 78 postmortem examinations in patients with sudden infant death syndrome [10].

The challenge in differentiating metaphyseal variations from CMLs has been highlighted in several studies. Karmazyn et al. reported that following a double read of skeletal surveys, four of 19 CMLs diagnosed by outside radiologists were read as metaphyseal variants by a pediatric radiologist [14]. Kleinman et al. reported that in one child metaphyseal fragmentation was misinterpreted as a CML, prompting a child abuse evaluation including skeletal survey [9]. A similar misdiagnosis was documented in another case report [15].

Our study is the first to evaluate the accuracy of radiologists in differentiating between CMLs and metaphyseal variations. Our study is focused on the knees, as from our experience this is both the most common location where metaphyseal variations are misinterpreted as CMLs and also the most common location of CMLs [16].

On average, the agreement between radiologists in our study was substantial (kappa = 0.61) with no significant difference in the rate of low confidence diagnosis between pediatric and adult radiologists. The most experienced pair of pediatric radiologists had the highest agreement (kappa = 0.84).

Both groups of radiologists had high specificity (90.9%) and PPV (95.6%) with no significant difference between them. These findings suggest that when radiologists diagnose CMLs, there is a high probability that the diagnosis is correct. However, there were moderate sensitivity (74.9%) and NPV (57.6%). The pediatric radiologists performed significantly better than the adult radiologists in both sensitivity and NPV, suggesting they may be better at detection of CMLs.

Diagnostic performance was significantly better at the initial radiographs compared to the follow-up radiographs, with higher accuracy, sensitivity, and NPV, whereas specificity and PPV did not show a significant difference. This suggests that the ability to reliably diagnose CML was better on the initial compared to follow-up radiographs. The reduced accuracy on follow-ups can be explained by healing and normalization of the CML on follow-up radiographs [17].

Metaphyseal fragmentation was the most frequently identified metaphyseal variation. Compared with the study of Kleinman et al. [9], our study shows that this variation also occurs in infants. In contrast to CMLs, which in about two-thirds involved the entire metaphysis or at least three metaphyseal locations, metaphyseal fragmentation involved only the medial and/or posterior metaphyses in 238 of 249 (95.6%) femurs and all 60 (100%) tibias.

In the CML group, four children were initially diagnosed with metaphyseal variation and five initially diagnosed with normal metaphyses were diagnosed on follow-up radiographs as CML, emphasizing the importance of follow-up radiographs. In a study by Kleinman et al., most fractures newly identified on follow-up skeletal survey involved CMLs and ribs [18]. In the metaphyseal variation group, only one adult radiologist diagnosed CML on follow-up radiographs, suggesting that metaphyseal variations diagnosed on initial radiographs are unlikely to be diagnosed as a CML on follow-up skeletal survey.

CML is considered highly specific for child abuse. Therefore, its presence should raise suspicion for abuse, even if it is the only detected lesion [3]. However, CMLs rarely occur in isolation without other fractures [7, 15]. Notably, of children in the metaphyseal variation group in our study, about a third had only a skull fracture while the remainder had no other fractures. Radiologists should be especially prudent when metaphyseal changes appear in isolation, as misinterpreting metaphyseal variations as CMLs could significantly impact both medical and child protection management.

Our study has several limitations. There is no gold standard for the diagnosis of CML and metaphyseal variations. Instead, cases are diagnosed based on consensus review by two experienced radiologists. It is likely that metaphyseal variations are underrepresented, as some may not have been mentioned in radiology reports—particularly by experienced radiologists who may have regarded them as normal findings. Additionally, only five children in the metaphyseal variation group had follow-up radiographs, potentially limiting our assessment of change in radiologists’ interpretation between initial and follow-up radiographs. In addition, radiologists were blinded to prior radiographs and ultrasound studies that are occasionally used in challenging cases, which could result in the incorrect diagnosis of healing CMLs as metaphyseal variations or as normal on follow-up radiographs.

In summary, we found substantial agreement between radiologists in the evaluation of CMLs and metaphyseal variations of the distal femur and proximal tibia. Radiologists demonstrated high specificity and positive predictive value in diagnosing CMLs and distinguishing them from metaphyseal variations. Pediatric radiologists performed significantly better than adult radiologists in both sensitivity and negative predictive value, suggesting superior ability in detecting CMLs.

Metaphyseal fragmentation was the most common metaphyseal variation, predominantly located in the medial and posterior metaphysis of the distal femur. Importantly, metaphyseal variations identified on initial radiographs were unlikely to be later diagnosed as CMLs on follow-up imaging.

## Supplementary Information

Below is the link to the electronic supplementary material.

ESM 1 (DOCX 18.1 KB)

ESM 2 (DOCX 25.7 KB)

ESM 3 (DOCX 28.0 KB)

## Data Availability

No datasets were generated or analysed during the current study.
