# Supplementary material for: Can metaphyseal variations in the distal femurs and proximal tibias be distinguished from classic metaphyseal lesions?
Source: Pediatr Radiol. 2025 Oct 1;55(13):2752–62. doi: 10.1007/s00247-025-06398-w (PMC12708683; doi:10.1007/s00247-025-06398-w)
Supplement: Supplementary file 1 — (DOCX 18.1 KB) [file 247_2025_6398_MOESM1_ESM.docx]

**Supplementary Table 1.**  Summary of injuries found on physical examination and imaging in classic metaphyseal lesions (CMLs) and metaphyseal variations groups. N (%, 95% CI for %).

|  | CML (n=44) | MV(n=22) | P- values |
| --- | --- | --- | --- |
| Physical examination- any injury | 29 (65.9%, 20.5%-49.9%) | 17 (77.3%, 7.8%-45.4%) | <0.001 |
| Bruising | 25 (56.8%, 41.0%-71.7%) | 1 (4.5%, 0.1%-22.8%) | <0.001 |
| Burn injury | 18 (40.9%, 26.3%-56.8%) | 0 (0%, 0%-15.4%) | 1.000 |
| Retinal bleeding | 1 (2.3%, 0.1%-12.0%) | 0 (0%, 0%-15.4%) | 0.545 |
| Subconjunctival H | 3 (6.8%, 1.4%-18.7%) | 0 (0%, 0%-15.4%) | 0.545 |
| Torn frenulum | 3 (6.8%, 1.4%-18.7%) | 0 (0%, 0%-15.4%) | 0.655 |
| Head trauma | 5 (11.4%, 3.8%-24.6%) | 1 (4.5%, 0.1%-22.8%) | 0.310 |
| Subdural | 10 (22.7%, 11.5%-37.8%) | 2 (9.1%, 1.1%-29.2%) | 0.312 |
| Parenchymal contusion | 9 (20.5%, 9.8%-35.3%) | 2 (9.1%, 1.1%-29.2%) | 0.545 |
| Parenchymal bleeding | 3 (6.8%, 1.4%-18.7%) | 0 (0%, 0%-15.4%) | 1.000 |
| Acute ischemia | 1 (2.3%, 0.1%-12.0%) | 0 (0%, 0%-15.4%) | 0.545 |
| Venous thrombosis | 3 (6.8%, 1.4%-18.7%) | 0 (0%, 0%-15.4%) | 1.000 |
| Fractures | 1 (2.3%, 0.1%-12.0%) | 0 (0%, 0%-15.4%) | <0.001 |
| Skull | 41 (93.2%, 81.3%-98.6%) | 7 (31.8%, 13.9%-54.9%) | 0.012 |
| Ribs | 3 (6.8%, 1.4%-18.7%) | 7 (31.8%, 13.9%-54.9%) | <0.001 |
| Upper extremity | 21 (47.7%, 32.5%-63.3%) | 0 (0%, 0%-15.4%) | <0.001 |
| Lower extremity | 23 (52.3%, 36.7%-67.5%) | 0 (0%, 0%-15.4%) | <0.001 |
| Spine | 31 (70.5%, 54.8%-83.2%) | 0 (0%, 0%-15.4%) | 1.000 |
| Hands | 0 (0%, 0%-8.0%) | 0 (0%, 0%-15.4%) | 0.549 |
| Feet | 2 (4.5%, 0.6%-15.5%) | 0 (0%, 0%-15.4%) | 1.000 |
| Pelvis | 1 (2.3%, 0.1%-12.0%) | 0 (0%, 0%-15.4%) | 1.000 |
| Others | 1 (2.3%, 0.1%-12.0%) | 0 (0%, 0%-15.4%) | 0.167 |

CML- classic metaphyseal lesions, MV- metaphyseal variation, PE- physical examination, H- hemorrhage
