# Supplementary material for: Can metaphyseal variations in the distal femurs and proximal tibias be distinguished from classic metaphyseal lesions?
Source: Pediatr Radiol. 2025 Oct 1;55(13):2752–62. doi: 10.1007/s00247-025-06398-w (PMC12708683; doi:10.1007/s00247-025-06398-w)
Supplement: Supplementary file 2 — (DOCX 25.7 KB) [file 247_2025_6398_MOESM2_ESM.docx]

**Supplementary Table 2.** Location of the radiographic signs within the metaphysis in the femurs. N (%, 95% CI for %).

|  |  |  | One location | | | | Two locations | | | | | | ≥ 3 locations |
| --- | --- | --- | --- | --- | --- | --- | --- | --- | --- | --- | --- | --- | --- |
| Radiographic sign | Examiner | N | Anterior | Posterior | Lateral | Medial | Anterior medial | Anterior lateral | Posterior medial | Posterior lateral | Anterior posterior | Medial lateral |  |
| Spur | All | 60 | 3 (5.0%, 1.0%-13.9%) | 0 (0%, 0%-6.0%) | 37 (61.7%, 48.2%-73.9%) | 4 (6.7%, 1.8%-16.2%) | 0 (0%, 0%-6.0%) | 9 (15.0%, 7.1%-26.6%) | 2 (3.3%, 0.4%-11.5%) | 0 (0%, 0%-6.0%) | 0 (0%, 0%-6.0%) | 4 (6.7%, 1.8%-16.2%) | 1 (1.7%, <0.1%-8.9%) |
|  | Pediatric | 26 | 3 (11.5%, 2.4%-30.2%) | 0 (0%, 0%-13.2%) | 15 (57.7%, 36.9%-76.6%) | 2 (7.7%, 0.9%-25.1%) | 0 (0%, 0%-13.2%) | 6 (23.1%, 9.0%-43.6%) | 0 (0%, 0%-13.2%) | 0 (0%, 0%-13.2%) | 0 (0%, 0%-13.2%) | 0 (0%, 0%-13.2%) | 0 (0%, 0%-13.2%) |
|  | Adult | 34 | 0 (0%, 0%-10.3%) | 0 (0%, 0%-10.3%) | 22 (64.7%, 46.5%-80.3%) | 2 (5.9%, 0.7%-19.7%) | 0 (0%, 0%-10.3%) | 3 (8.8%, 1.9%-23.7%) | 2 (5.9%, 0.7%-19.7%) | 0 (0%, 0%-10.3%) | 0 (0%, 0%-10.3%) | 4 (11.8%, 3.3%-27.5%) | 1 (2.9%, 0.1%-15.3%) |
| Step Off | All | 35 | 0 (0%, 0%-10.0%) | 7 (20.0%, 8.4%-36.9%) | 12 (34.3%, 19.1%-52.2%) | 11 (31.4%, 16.9%-49.3%) | 0 (0%, 0%-10.0%) | 0 (0%, 0%-10.0%) | 3 (8.6%, 1.8%-23.1%) | 1 (2.9%, 0.1%-14.9%) | 0 (0%, 0%-10.0%) | 1 (2.9%, 0.1%-14.9%) | 0 (0%, 0%-10.0%) |
|  | Pediatric | 18 | 0 (0%, 0%-18.5%) | 2 (11.1%, 1.4%-34.7%) | 8 (44.4%, 21.5%-69.2%) | 7 (38.9%, 17.3%-64.3%) | 0 (0%, 0%-18.5%) | 0 (0%, 0%-18.5%) | 1 (5.6%, 0.1%-27.3%) | 0 (0%, 0%-18.5%) | 0 (0%, 0%-18.5%) | 0 (0%, 0%-18.5%) | 0 (0%, 0%-18.5%) |
|  | Adult | 17 | 0 (0%, 0%-19.5%) | 5 (29.4%, 10.3%-56.0%) | 4 (23.5%, 6.8%-49.9%) | 4 (23.5%, 6.8%-49.9%) | 0 (0%, 0%-19.5%) | 0 (0%, 0%-19.5%) | 2 (11.8%, 1.5%-36.4%) | 1 (5.9%, 0.1%-28.7%) | 0 (0%, 0%-19.5%) | 1 (5.9%, 0.1%-28.7%) | 0 (0%, 0%-19.5%) |
| Fragmentation | All | 249 | 0 (0%, 0%-1.5%) | 112 (45.0%, 38.7%-51.4%) | 2 (0.8%, 0.1%-2.9%) | 40 (16.1%, 11.7%-21.2%) | 0 (0%, 0%-1.5%) | 0 (0%, 0%-1.5%) | 86 (34.5%, 28.6%-40.8%) | 6 (2.4%, 0.9%-5.2%) | 0 (0%, 0%-1.5%) | 3 (1.2%, 0.2%-3.5%) | 0 (0%, 0%-1.5%) |
|  | Pediatric | 140 | 0 (0%, 0%-2.6%) | 55 (39.3%, 31.1%-47.9%) | 2 (1.4%, 0.2%-5.1%) | 21 (15.0%, 9.5%-22.0%) | 0 (0%, 0%-2.6%) | 0 (0%, 0%-2.6%) | 58 (41.4%, 33.2%-50.1%) | 2 (1.4%, 0.2%-5.1%) | 0 (0%, 0%-2.6%) | 2 (1.4%, 0.2%-5.1%) | 0 (0%, 0%-2.6%) |
|  | Adult | 109 | 0 (0%, 0%-3.3%) | 57 (52.3%, 42.5%-61.9%) | 0 (0%, 0%-3.3%) | 19 (17.4%, 10.8%-25.9%) | 0 (0%, 0%-3.3%) | 0 (0%, 0%-3.3%) | 28 (25.7%, 17.8%-34.9%) | 4 (3.7%, 1.0%-9.1%) | 0 (0%, 0%-3.3%) | 1 (0.9%, <0.1%-5.0%) | 0 (0%, 0%-3.3%) |
| Corner Fracture | All | 194 | 4 (2.1%, 0.6%-5.2%) | 19 (9.8%, 6.0%-14.9%) | 18 (9.3%, 5.6%-14.3%) | 45 (23.2%, 17.5%-29.8%) | 0 (0%, 0%-1.9%) | 2 (1.0%, 0.1%-3.7%) | 22 (11.3%, 7.2%-16.7%) | 7 (3.6%, 1.5%-7.3%) | 2 (1.0%, 0.1%-3.7%) | 6 (3.1%, 1.1%-6.6%) | 69 (35.6%, 28.8%-42.7%) |
|  | Pediatric | 112 | 4 (3.6%, 1.0%-8.9%) | 7 (6.3%, 2.5%-12.5%) | 9 (8.0%, 3.7%-14.7%) | 15 (13.4%, 7.7%-21.1%) | 0 (0%, 0%-3.2%) | 2 (1.8%, 0.2%-6.3%) | 14 (12.5%, 7.0%-20.1%) | 3 (2.7%, 0.6%-7.6%) | 0 (0%, 0%-3.2%) | 1 (0.9%, <0.1%-4.9%) | 57 (50.9%, 41.3%-60.5%) |
|  | Adult | 82 | 0 (0%, 0%-4.4%) | 12 (14.6%, 7.8%-24.2%) | 9 (11.0%, 5.1%-19.8%) | 30 (36.6%, 26.2%-48.0%) | 0 (0%, 0%-4.4%) | 0 (0%, 0%-4.4%) | 8 (9.8%, 4.3%-18.3%) | 4 (4.9%, 1.3%-12.0%) | 2 (2.4%, 0.3%-8.5%) | 5 (6.1%, 2.0%-13.7%) | 12 (14.6%, 7.8%-24.2%) |
| Bucket Handle | All | 137 | 2 (1.5%, 0.2%-5.2%) | 4 (2.9%, 0.8%-7.3%) | 10 (7.3%, 3.6%-13.0%) | 4 (2.9%, 0.8%-7.3%) | 0 (0%, 0%-2.7%) | 0 (0%, 0%-2.7%) | 0 (0%, 0%-2.7%) | 0 (0%, 0%-2.7%) | 0 (0%, 0%-2.7%) | 0 (0%, 0%-2.7%) | 117 (85.4%, 78.4%-90.8%) |
|  | Pediatric | 88 | 1 (1.1%, <0.1%-6.2%) | 2 (2.3%, 0.3%-8.0%) | 2 (2.3%, 0.3%-8.0%) | 0 (0%, 0%-4.1%) | 0 (0%, 0%-4.1%) | 0 (0%, 0%-4.1%) | 0 (0%, 0%-4.1%) | 0 (0%, 0%-4.1%) | 0 (0%, 0%-4.1%) | 0 (0%, 0%-4.1%) | 83 (94.3%, 87.2%-98.1%) |
|  | Adult | 49 | 1 (2.0%, 0.1%-10.9%) | 2 (4.1%, 0.5%-14.0%) | 8 (16.3%, 7.3%-29.7%) | 4 (8.2%, 2.3%-19.6%) | 0 (0%, 0%-7.3%) | 0 (0%, 0%-7.3%) | 0 (0%, 0%-7.3%) | 0 (0%, 0%-7.3%) | 0 (0%, 0%-7.3%) | 0 (0%, 0%-7.3%) | 34 (69.4%, 54.6%-81.7%) |
| Subphyseal Lucency | All | 38 | 0 (0%, 0%-9.3%) | 0 (0%, 0%-9.3%) | 8 (21.1%, 9.6%-37.3%) | 14 (36.8%, 21.8%-54.0%) | 0 (0%, 0%-9.3%) | 0 (0%, 0%-9.3%) | 1 (2.6%, 0.1%-13.8%) | 0 (0%, 0%-9.3%) | 0 (0%, 0%-9.3%) | 0 (0%, 0%-9.3%) | 15 (39.5%, 24.0%-56.6%) |
|  | Pediatric | 24 | 0 (0%, 0%-14.2%) | 0 (0%, 0%-14.2%) | 4 (16.7%, 4.7%-37.4%) | 7 (29.2%, 12.6%-51.1%) | 0 (0%, 0%-14.2%) | 0 (0%, 0%-14.2%) | 0 (0%, 0%-14.2%) | 0 (0%, 0%-14.2%) | 0 (0%, 0%-14.2%) | 0 (0%, 0%-14.2%) | 13 (54.2%, 32.8%-74.4%) |
|  | Adult | 14 | 0 (0%, 0%-23.2%) | 0 (0%, 0%-23.2%) | 4 (28.6%, 8.4%-58.1%) | 7 (50.0%, 23.0%-77.0%) | 0 (0%, 0%-23.2%) | 0 (0%, 0%-23.2%) | 1 (7.1%, 0.2%-33.9%) | 0 (0%, 0%-23.2%) | 0 (0%, 0%-23.2%) | 0 (0%, 0%-23.2%) | 2 (14.3%, 1.8%-42.8%) |
| Deformed Corner | All | 64 | 0 (0%, 0%-5.6%) | 5 (7.8%, 2.6%-17.3%) | 5 (7.8%, 2.6%-17.3%) | 26 (40.6%, 28.5%-53.6%) | 0 (0%, 0%-5.6%) | 0 (0%, 0%-5.6%) | 0 (0%, 0%-5.6%) | 0 (0%, 0%-5.6%) | 0 (0%, 0%-5.6%) | 2 (3.1%, 0.4%-10.8%) | 26 (40.6%, 28.5%-53.6%) |
|  | Pediatric | 50 | 0 (0%, 0%-7.1%) | 2 (4.0%, 0.5%-13.7%) | 5 (10.0%, 3.3%-21.8%) | 18 (36.0%, 22.9%-50.8%) | 0 (0%, 0%-7.1%) | 0 (0%, 0%-7.1%) | 0 (0%, 0%-7.1%) | 0 (0%, 0%-7.1%) | 0 (0%, 0%-7.1%) | 1 (2.0%, 0.1%-10.6%) | 24 (48.0%, 33.7%-62.6%) |
|  | Adult | 14 | 0 (0%, 0%-23.2%) | 3 (21.4%, 4.7%-50.8%) | 0 (0%, 0%-23.2%) | 8 (57.1%, 28.9%-82.3%) | 0 (0%, 0%-23.2%) | 0 (0%, 0%-23.2%) | 0 (0%, 0%-23.2%) | 0 (0%, 0%-23.2%) | 0 (0%, 0%-23.2%) | 1 (7.1%, 0.2%-33.9%) | 2 (14.3%, 1.8%-42.8%) |
| Metaphyseal Irregularity | All | 123 | 2 (1.6%, 0.2%-5.8%) | 2 (1.6%, 0.2%-5.8%) | 3 (2.4%, 0.5%-7.0%) | 8 (6.5%, 2.8%-12.4%) | 0 (0%, 0%-3.0%) | 0 (0%, 0%-3.0%) | 0 (0%, 0%-3.0%) | 1 (0.8%, <0.1%-4.4%) | 1 (0.8%, <0.1%-4.4%) | 2 (1.6%, 0.2%-5.8%) | 104 (84.6%, 76.9%-90.4%) |
|  | Pediatric | 75 | 2 (2.7%, 0.3%-9.3%) | 1 (1.3%, <0.1%-7.2%) | 3 (4.0%, 0.8%-11.2%) | 7 (9.3%, 3.8%-18.3%) | 0 (0%, 0%-4.8%) | 0 (0%, 0%-4.8%) | 0 (0%, 0%-4.8%) | 0 (0%, 0%-4.8%) | 0 (0%, 0%-4.8%) | 2 (2.7%, 0.3%-9.3%) | 60 (80.0%, 69.2%-88.4%) |
|  | Adult | 48 | 0 (0%, 0%-7.4%) | 1 (2.1%, 0.1%-11.1%) | 0 (0%, 0%-7.4%) | 1 (2.1%, 0.1%-11.1%) | 0 (0%, 0%-7.4%) | 0 (0%, 0%-7.4%) | 0 (0%, 0%-7.4%) | 1 (2.1%, 0.1%-11.1%) | 1 (2.1%, 0.1%-11.1%) | 0 (0%, 0%-7.4%) | 44 (91.7%, 80.0%-97.7%) |
| Subperiosteal New Bone Formation | All | 83 | 6 (7.2%, 2.7%-15.1%) | 4 (4.8%, 1.3%-11.9%) | 6 (7.2%, 2.7%-15.1%) | 2 (2.4%, 0.3%-8.4%) | 0 (0%, 0%-4.3%) | 1 (1.2%, <0.1%-6.5%) | 0 (0%, 0%-4.3%) | 0 (0%, 0%-4.3%) | 1 (1.2%, <0.1%-6.5%) | 0 (0%, 0%-4.3%) | 63 (75.9%, 65.3%-84.6%) |
|  | Pediatric | 62 | 6 (9.7%, 3.6%-19.9%) | 3 (4.8%, 1.0%-13.5%) | 6 (9.7%, 3.6%-19.9%) | 1 (1.6%, <0.1%-8.7%) | 0 (0%, 0%-5.8%) | 1 (1.6%, <0.1%-8.7%) | 0 (0%, 0%-5.8%) | 0 (0%, 0%-5.8%) | 0 (0%, 0%-5.8%) | 0 (0%, 0%-5.8%) | 45 (72.6%, 59.8%-83.1%) |
|  | Adult | 21 | 0 (0%, 0%-16.1%) | 1 (4.8%, 0.1%-23.8%) | 0 (0%, 0%-16.1%) | 1 (4.8%, 0.1%-23.8%) | 0 (0%, 0%-16.1%) | 0 (0%, 0%-16.1%) | 0 (0%, 0%-16.1%) | 0 (0%, 0%-16.1%) | 1 (4.8%, 0.1%-23.8%) | 0 (0%, 0%-16.1%) | 18 (85.7%, 63.7%-97.0%) |

N- Overall number of femurs reviewed by all 8 radiologists and 4 pediatric and adult radiologists when the radiologist indicated a) presence of only 1 sign or b) presence of multiple signs but all signs had the same single location identifier specified.
