# Supplementary material for: Can metaphyseal variations in the distal femurs and proximal tibias be distinguished from classic metaphyseal lesions?
Source: Pediatr Radiol. 2025 Oct 1;55(13):2752–62. doi: 10.1007/s00247-025-06398-w (PMC12708683; doi:10.1007/s00247-025-06398-w)
Supplement: Supplementary file 3 — (DOCX 28.0 KB) [file 247_2025_6398_MOESM3_ESM.docx]

**Supplementary Table 3.** Location of the radiographic signs within the metaphysis in the tibias. N (%, 95% CI for %).

|  |  |  | One location | | | | Two locations | | | | | | ≥ 3 locations |
| --- | --- | --- | --- | --- | --- | --- | --- | --- | --- | --- | --- | --- | --- |
| Radiographic sign | Examiner | N | Anterior | Posterior | Lateral | Medial | Anterior medial | Anterior lateral | Posterior medial | Posterior lateral | Anterior posterior | Medial lateral |  |
| Spur | All | 3 | 0 (0%, 0%-70.8%) | 0 (0%, 0%-70.8%) | 0 (0%, 0%-70.8%) | 1 (33.3%, 0.8%-90.6%) | 0 (0%, 0%-70.8%) | 0 (0%, 0%-70.8%) | 1 (33.3%, 0.8%-90.6%) | 0 (0%, 0%-70.8%) | 0 (0%, 0%-70.8%) | 0 (0%, 0%-70.8%) | 1 (33.3%, 0.8%-90.6%) |
|  | Pediatric | 2 | 0 (0%, 0%-84.2%) | 0 (0%, 0%-84.2%) | 0 (0%, 0%-84.2%) | 1 (50.0%, 1.3%-98.7%) | 0 (0%, 0%-84.2%) | 0 (0%, 0%-84.2%) | 1 (50.0%, 1.3%-98.7%) | 0 (0%, 0%-84.2%) | 0 (0%, 0%-84.2%) | 0 (0%, 0%-84.2%) | 0 (0%, 0%-84.2%) |
|  | Adult | 1 | 0 (0%, 0%-97.5%) | 0 (0%, 0%-97.5%) | 0 (0%, 0%-97.5%) | 0 (0%, 0%-97.5%) | 0 (0%, 0%-97.5%) | 0 (0%, 0%-97.5%) | 0 (0%, 0%-97.5%) | 0 (0%, 0%-97.5%) | 0 (0%, 0%-97.5%) | 0 (0%, 0%-97.5%) | 1 (100%, 2.5%-100%) |
| Step Off | All | 6 | 0 (0%, 0%-45.9%) | 0 (0%, 0%-45.9%) | 0 (0%, 0%-45.9%) | 6 (100%, 54.1%-100%) | 0 (0%, 0%-45.9%) | 0 (0%, 0%-45.9%) | 0 (0%, 0%-45.9%) | 0 (0%, 0%-45.9%) | 0 (0%, 0%-45.9%) | 0 (0%, 0%-45.9%) | 0 (0%, 0%-45.9%) |
|  | Pediatric | 4 | 0 (0%, 0%-60.2%) | 0 (0%, 0%-60.2%) | 0 (0%, 0%-60.2%) | 4 (100%, 39.8%-100%) | 0 (0%, 0%-60.2%) | 0 (0%, 0%-60.2%) | 0 (0%, 0%-60.2%) | 0 (0%, 0%-60.2%) | 0 (0%, 0%-60.2%) | 0 (0%, 0%-60.2%) | 0 (0%, 0%-60.2%) |
|  | Adult | 2 | 0 (0%, 0%-84.2%) | 0 (0%, 0%-84.2%) | 0 (0%, 0%-84.2%) | 2 (100%, 15.8%-100%) | 0 (0%, 0%-84.2%) | 0 (0%, 0%-84.2%) | 0 (0%, 0%-84.2%) | 0 (0%, 0%-84.2%) | 0 (0%, 0%-84.2%) | 0 (0%, 0%-84.2%) | 0 (0%, 0%-84.2%) |
| Fragmentation | All | 60 | 0 (0%, 0%-6.0%) | 1 (1.7%, <0.1%-8.9%) | 0 (0%, 0%-6.0%) | 54 (90.0%, 79.5%-96.2%) | 0 (0%, 0%-6.0%) | 0 (0%, 0%-6.0%) | 5 (8.3%, 2.8%-18.4%) | 0 (0%, 0%-6.0%) | 0 (0%, 0%-6.0%) | 0 (0%, 0%-6.0%) | 0 (0%, 0%-6.0%) |
|  | Pediatric | 42 | 0 (0%, 0%-8.4%) | 1 (2.4%, 0.1%-12.6%) | 0 (0%, 0%-8.4%) | 36 (85.7%, 71.5%-94.6%) | 0 (0%, 0%-8.4%) | 0 (0%, 0%-8.4%) | 5 (11.9%, 4.0%-25.6%) | 0 (0%, 0%-8.4%) | 0 (0%, 0%-8.4%) | 0 (0%, 0%-8.4%) | 0 (0%, 0%-8.4%) |
|  | Adult | 18 | 0 (0%, 0%-18.5%) | 0 (0%, 0%-18.5%) | 0 (0%, 0%-18.5%) | 18 (100%, 81.5%-100%) | 0 (0%, 0%-18.5%) | 0 (0%, 0%-18.5%) | 0 (0%, 0%-18.5%) | 0 (0%, 0%-18.5%) | 0 (0%, 0%-18.5%) | 0 (0%, 0%-18.5%) | 0 (0%, 0%-18.5%) |
| Corner Fracture | All | 97 | 0 (0%, 0%-3.7%) | 8 (8.2%, 3.6%-15.6%) | 0 (0%, 0%-3.7%) | 26 (26.8%, 18.3%-36.8%) | 1 (1.0%, <0.1%-5.6%) | 0 (0%, 0%-3.7%) | 16 (16.5%, 9.7%-25.4%) | 0 (0%, 0%-3.7%) | 7 (7.2%, 3.0%-14.3%) | 0 (0%, 0%-3.7%) | 39 (40.2%, 30.4%-50.7%) |
|  | Pediatric | 55 | 0 (0%, 0%-6.5%) | 2 (3.6%, 0.4%-12.5%) | 0 (0%, 0%-6.5%) | 9 (16.4%, 7.8%-28.8%) | 1 (1.8%, <0.1%-9.7%) | 0 (0%, 0%-6.5%) | 6 (10.9%, 4.1%-22.2%) | 0 (0%, 0%-6.5%) | 3 (5.5%, 1.1%-15.1%) | 0 (0%, 0%-6.5%) | 34 (61.8%, 47.7%-74.6%) |
|  | Adult | 42 | 0 (0%, 0%-8.4%) | 6 (14.3%, 5.4%-28.5%) | 0 (0%, 0%-8.4%) | 17 (40.5%, 25.6%-56.7%) | 0 (0%, 0%-8.4%) | 0 (0%, 0%-8.4%) | 10 (23.8%, 12.1%-39.5%) | 0 (0%, 0%-8.4%) | 4 (9.5%, 2.7%-22.6%) | 0 (0%, 0%-8.4%) | 5 (11.9%, 4.0%-25.6%) |
| Bucket Handle | All | 128 | 0 (0%, 0%-2.8%) | 2 (1.6%, 0.2%-5.5%) | 1 (0.8%, <0.1%-4.3%) | 12 (9.4%, 4.9%-15.8%) | 0 (0%, 0%-2.8%) | 0 (0%, 0%-2.8%) | 0 (0%, 0%-2.8%) | 0 (0%, 0%-2.8%) | 0 (0%, 0%-2.8%) | 0 (0%, 0%-2.8%) | 113 (88.3%, 81.4%-93.3%) |
|  | Pediatric | 82 | 0 (0%, 0%-4.4%) | 2 (2.4%, 0.3%-8.5%) | 0 (0%, 0%-4.4%) | 6 (7.3%, 2.7%-15.2%) | 0 (0%, 0%-4.4%) | 0 (0%, 0%-4.4%) | 0 (0%, 0%-4.4%) | 0 (0%, 0%-4.4%) | 0 (0%, 0%-4.4%) | 0 (0%, 0%-4.4%) | 74 (90.2%, 81.7%-95.7%) |
|  | Adult | 46 | 0 (0%, 0%-7.7%) | 0 (0%, 0%-7.7%) | 1 (2.2%, 0.1%-11.5%) | 6 (13.0%, 4.9%-26.3%) | 0 (0%, 0%-7.7%) | 0 (0%, 0%-7.7%) | 0 (0%, 0%-7.7%) | 0 (0%, 0%-7.7%) | 0 (0%, 0%-7.7%) | 0 (0%, 0%-7.7%) | 39 (84.8%, 71.1%-93.7%) |
| Subphyseal Lucency | All | 29 | 0 (0%, 0%-11.9%) | 4 (13.8%, 3.9%-31.7%) | 0 (0%, 0%-11.9%) | 6 (20.7%, 8.0%-39.7%) | 0 (0%, 0%-11.9%) | 0 (0%, 0%-11.9%) | 0 (0%, 0%-11.9%) | 0 (0%, 0%-11.9%) | 0 (0%, 0%-11.9%) | 0 (0%, 0%-11.9%) | 19 (65.5%, 45.7%-82.1%) |
|  | Pediatric | 19 | 0 (0%, 0%-17.6%) | 2 (10.5%, 1.3%-33.1%) | 0 (0%, 0%-17.6%) | 2 (10.5%, 1.3%-33.1%) | 0 (0%, 0%-17.6%) | 0 (0%, 0%-17.6%) | 0 (0%, 0%-17.6%) | 0 (0%, 0%-17.6%) | 0 (0%, 0%-17.6%) | 0 (0%, 0%-17.6%) | 15 (78.9%, 54.4%-93.9%) |
|  | Adult | 10 | 0 (0%, 0%-30.8%) | 2 (20.0%, 2.5%-55.6%) | 0 (0%, 0%-30.8%) | 4 (40.0%, 12.2%-73.8%) | 0 (0%, 0%-30.8%) | 0 (0%, 0%-30.8%) | 0 (0%, 0%-30.8%) | 0 (0%, 0%-30.8%) | 0 (0%, 0%-30.8%) | 0 (0%, 0%-30.8%) | 4 (40.0%, 12.2%-73.8%) |
| Deformed Corner | All | 46 | 0 (0%, 0%-7.7%) | 3 (6.5%, 1.4%-17.9%) | 0 (0%, 0%-7.7%) | 24 (52.2%, 36.9%-67.1%) | 0 (0%, 0%-7.7%) | 0 (0%, 0%-7.7%) | 0 (0%, 0%-7.7%) | 0 (0%, 0%-7.7%) | 0 (0%, 0%-7.7%) | 0 (0%, 0%-7.7%) | 19 (41.3%, 27.0%-56.8%) |
|  | Pediatric | 41 | 0 (0%, 0%-8.6%) | 3 (7.3%, 1.5%-19.9%) | 0 (0%, 0%-8.6%) | 19 (46.3%, 30.7%-62.6%) | 0 (0%, 0%-8.6%) | 0 (0%, 0%-8.6%) | 0 (0%, 0%-8.6%) | 0 (0%, 0%-8.6%) | 0 (0%, 0%-8.6%) | 0 (0%, 0%-8.6%) | 19 (46.3%, 30.7%-62.6%) |
|  | Adult | 5 | 0 (0%, 0%-52.2%) | 0 (0%, 0%-52.2%) | 0 (0%, 0%-52.2%) | 5 (100%, 47.8%-100%) | 0 (0%, 0%-52.2%) | 0 (0%, 0%-52.2%) | 0 (0%, 0%-52.2%) | 0 (0%, 0%-52.2%) | 0 (0%, 0%-52.2%) | 0 (0%, 0%-52.2%) | 0 (0%, 0%-52.2%) |
| Metaphyseal Irregularity | All | 134 | 1 (0.7%, <0.1%-4.1%) | 4 (3.0%, 0.8%-7.5%) | 1 (0.7%, <0.1%-4.1%) | 18 (13.4%, 8.2%-20.4%) | 1 (0.7%, <0.1%-4.1%) | 0 (0%, 0%-2.7%) | 1 (0.7%, <0.1%-4.1%) | 0 (0%, 0%-2.7%) | 0 (0%, 0%-2.7%) | 2 (1.5%, 0.2%-5.3%) | 106 (79.1%, 71.2%-85.6%) |
|  | Pediatric | 101 | 1 (1.0%, <0.1%-5.4%) | 3 (3.0%, 0.6%-8.4%) | 1 (1.0%, <0.1%-5.4%) | 17 (16.8%, 10.1%-25.6%) | 1 (1.0%, <0.1%-5.4%) | 0 (0%, 0%-3.6%) | 1 (1.0%, <0.1%-5.4%) | 0 (0%, 0%-3.6%) | 0 (0%, 0%-3.6%) | 2 (2.0%, 0.2%-7.0%) | 75 (74.3%, 64.6%-82.4%) |
|  | Adult | 33 | 0 (0%, 0%-10.6%) | 1 (3.0%, 0.1%-15.8%) | 0 (0%, 0%-10.6%) | 1 (3.0%, 0.1%-15.8%) | 0 (0%, 0%-10.6%) | 0 (0%, 0%-10.6%) | 0 (0%, 0%-10.6%) | 0 (0%, 0%-10.6%) | 0 (0%, 0%-10.6%) | 0 (0%, 0%-10.6%) | 31 (93.9%, 79.8%-99.3%) |
| Subperiosteal New Bone Formation | All | 55 | 0 (0%, 0%-6.5%) | 2 (3.6%, 0.4%-12.5%) | 1 (1.8%, <0.1%-9.7%) | 1 (1.8%, <0.1%-9.7%) | 0 (0%, 0%-6.5%) | 0 (0%, 0%-6.5%) | 0 (0%, 0%-6.5%) | 1 (1.8%, <0.1%-9.7%) | 0 (0%, 0%-6.5%) | 0 (0%, 0%-6.5%) | 50 (90.9%, 80.0%-97.0%) |
|  | Pediatric | 46 | 0 (0%, 0%-7.7%) | 2 (4.3%, 0.5%-14.8%) | 1 (2.2%, 0.1%-11.5%) | 0 (0%, 0%-7.7%) | 0 (0%, 0%-7.7%) | 0 (0%, 0%-7.7%) | 0 (0%, 0%-7.7%) | 1 (2.2%, 0.1%-11.5%) | 0 (0%, 0%-7.7%) | 0 (0%, 0%-7.7%) | 42 (91.3%, 79.2%-97.6%) |
|  | Adult | 9 | 0 (0%, 0%-33.6%) | 0 (0%, 0%-33.6%) | 0 (0%, 0%-33.6%) | 1 (11.1%, 0.3%-48.2%) | 0 (0%, 0%-33.6%) | 0 (0%, 0%-33.6%) | 0 (0%, 0%-33.6%) | 0 (0%, 0%-33.6%) | 0 (0%, 0%-33.6%) | 0 (0%, 0%-33.6%) | 8 (88.9%, 51.8%-99.7%) |

N- Overall number of tibias reviewed by all 8 radiologists and 4 pediatric and adult radiologists when the radiologist indicated a) presence of only 1 sign or b) presence of multiple signs but all signs had the same single location identifier specified.
